# Numerical and Experimental Analysis of DVA on the Flexible-Rigid Rail Vehicle Carbody Resonant Vibration

**DOI:** 10.3390/s22051922

**Published:** 2022-03-01

**Authors:** Sunil Kumar Sharma, Rakesh Chandmal Sharma, Jaesun Lee, Hong-Lae Jang

**Affiliations:** 1 School of Engineering & Applied Science, National Rail and Transportation Institute, Vadodara 390004, Gujarat, India; sk.sharma@nrti.edu.in; 2 Mechanical Engineering Department, Maharishi Markandeshwar, Mullana 133207, Haryana, India; drrcsharma@mmumullana.org; 3 School of Mechanical Engineering, Changwon National University, Changwon 51140, Korea; jaesun@changwon.ac.kr

**Keywords:** underframe equipment, suspension parameters, modal frequency, car-body bending frequency

## Abstract

This paper examines the influence of the equipment considered as a DVA (Dynamic Vibration Absorber) upon the mode of vertical vibrations of the car body in high-speed vehicles. The car body is represented as an Euler-Bernoulli beam to minimize flexible vibration. The DVA approach is used to find the appropriate suspension frequencies for various types of equipment. A vertical mathematical model with a flexible car body and equipment is developed to investigate the effect of equipment mass, suspension stiffness, damping, and mounting location on car-body flexible vibrations. A three-dimensional, rigid-flexible coupled vehicle system dynamics model is developed to simulate the car body and equipment’s response to track irregularities. The experimental result was considered to verify the theoretical analysis and dynamic simulation. The mathematical analysis demonstrates that the DVA theory can be used to design the suspension parameters of the equipment and that it is suitable and effective in reducing the flexible vibration of the car body in which the vertical bending mode is greatly affected. Heavy equipment should be mounted as close to the car body’s center as possible to achieve significant flexible vibration reduction, whereas light equipment contributes very little flexible vibration reduction.

## 1. Introduction

The vehicle vibrations due to track irregularities are considered when the vehicle ride quality is investigated. The track vibrations reach the car body (CB) due to irregularity inputs via rail wheel interaction, primary suspension system, and secondary suspension system. With the fast progress of high-speed rail vehicle technology, train operating speed increases while weight decreases to conserve energy. However, the high running speed expands the rail vehicle vibration frequency range, and the light structure allows the flexible modes to be more easily triggered by wheel/rail interaction [1]. It was found that the CB vibration is a more flexible movement compared to rigid movement. Therefore, to create innovative technologies to enhance the dynamic response of rail vehicles at higher speeds, there is a necessity to understand the function of CB flexibility [2].

Recent studies suggest that optimization design based on being lightweight may decrease cost, reduce the force between wheel and rail to protect them, achieve energy savings, and reduce emissions [3,4,5,6,7,8,9,10]. The stochastic vibration is obtained at the structure because the CB is adversely affected by track irregularities and wind pressure while the vehicle is moving. Low CB stiffness will reduce its natural frequency, leading to a low order natural frequency in the track’s excitation frequency range [11,12]. In this situation, the train will vibrate more, fatigue life will be shorter, and riding comfort will be less. As a result, the train’s operation quality will decrease significantly. Vibrations of the car body may be classified into two types: rigid and flexible. The rigid body modes that affect vertical ride comfort are bounce, pitch, and roll, which normally lie in a relatively low frequency range around 1 Hz [13,14]. The flexible modes are twisting and bending deformations of the car body. Typical flexible modes that affect ride comfort most are the first flexible modes that frequently occur close to the frequency range from 4 to 10 Hz, to which human beings tend to be sensitive [12,14].

Tomioka et al. [15] conducted extensive research on the structural dynamics of a rail vehicle’s CB. Through computations and observations, the investigations examine the structural flexibility of the CB and its effect on ride comfort. The energy and frequency bands of the track excitation generally rise as vehicle running speed increases, making them more ideal for delivering adequate energy to generate the flexural vibrations of the CB. Diana et al. [16] conducted a sensitivity analysis on the parameters that primarily influenced comfort performance for car bodies and discovered that specific track wavelengths and vehicle speeds could substantially impact the vibration levels of CB modes. Sun et al. [17] investigated the links between riding comfort and static displacement of suspension equipment. However, just a single device installed at the CB center was considered in those models. None of them offered a particular suspension technique [18,19,20,21,22,23].

However, rail vehicle coaches contain a variety of equipment under the chassis, which varies depending on the functional requirements of the coach [20,21,22,23,24,25,26,27,28,29,30,31,32,33,34,35,36]. As a result, the suspension characteristics of the equipment should be considered. Hence, this work aims to examine how resonant vibration of a flexible railway CB occurs. First, a 3D rigid-flexible coupled vehicle system dynamics model was created by combining MBS theory with FEM to simulate the behavior of the CB and equipment. A mathematical formulation was also developed to obtain the optimum suspension frequency of DVA. The experimental finding was used to validate the mathematical model and dynamic simulation. Then, the relationship between eigenmodes of the passenger CB and equipment under the chassis is analyzed. Finally, an optimized dynamic vibration absorber (DVA), taking account of the geometric filtering effect, is proposed to suppress the CB resonant vibration.

## 2. Mathematical Model of the Rigid-Flexible Coupled Vehicle System

**Figure 1:**
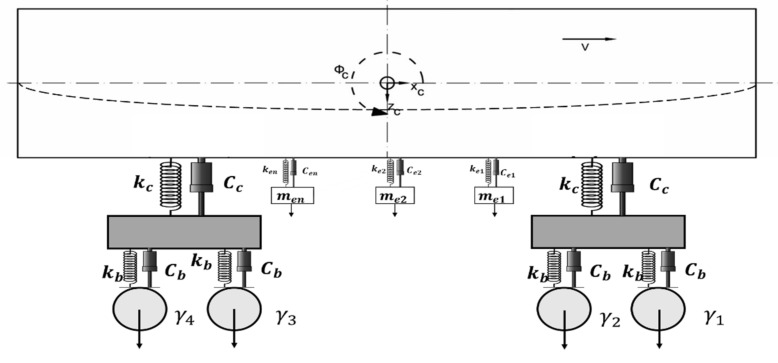
Schematic model of the passenger rail vehicle ICF coach.

A full-size ICF coach of an Indian railway is considered for the modeling and simulation analysis. The rail vehicle’s mathematical model includes the CB, two bogies, four-wheel sets, and suspended equipment, as shown in Figure 1. They are mechanically linked in the primary and secondary suspension systems via springs and dampers [37]. An Euler-Bernoulli type free-free equivalent beam describes the CB with a constant section and evenly distributed mass.

### 2.1. Modeling of Flexible Car Body

The CB is represented as a beam of Euler-Bernoulli type, with evenly distributed mass. The beam parameters are defined in terms of the vehicle’s body, where *m_c_* (37,960 kg) is the vehicle body mass, the elastic modulus is *E*, and the moment of inertia for the section is *I* (= 1,473,430 kg m^2^) [38]. The bounce and pitch CB vibration modes, as well as the first CB bending eigenmode in a vertical plane [39], are considered, and the vehicle body equation of motion for the general form is given in (Equation (1)).

(1) $$EI\frac{\partial^{4}w_{c}(x,t)}{\partial x^{4}}+\mu I\frac{\partial^{5}w_{c}\;(x,t\;)}{\partial x^{4}\partial t}+\rho_{c}\frac{\partial^{2}\;w_{c}(x,t)}{\partial t^{2}}=\;\sum_{i=1}^{2}\;F_{ci}\delta (x-l_{i})+\;\sum_{k=1}^{n}\;F_{ek}\;\delta (\;x-\;l_{ek})$$

where *δ* (.) is the Dirac delta function, *l_i_* is the distance (*I* = 1; 2), *F_ci_* stands for the forces derived from the secondary suspension corresponding to bogie *i*, and *F_ek_* represents the forces coming from the suspension of equipment (Equations (2) and (3)).

(2) $$F_{ci}=-2c_{c}\left(\frac{\partial w_{c}(l_{i},t)}{\partial t}-{\dot{z}}_{bi}\right)-2k_{c}\left(w_{c}(l_{i},t)-z_{bi}\right)$$

(3) $$F_{ek}=-2c_{ek}\left(\frac{\partial w_{c}(l_{ek},t)}{\partial t}-{\dot{z}}_{e}\right)-2k_{ek}\left(w_{c}(l_{ek},t)-z_{ek}\right)$$

where $c_{c}$ (0.035 MN-s/m) and $k_{c}$ (35 MN/m) are the damping and stiffness coefficients of the secondary suspension system [38]. The CB vertical movement *w_c_*(*x*; *t*) comes from the superposition of the two rigid vibration modes, namely bounce and pitch, with the first bending mode [39]:

(4) $$w_{c}\;(\;x,\;t\;)=\;z_{c}\;(t)\;+\;\left(\;x-\frac{L_{c}}{2}\right)\;\theta_{c}(t\;)+\;X_{c}\;(x)\;T_{c}(t\;)$$

where *T_c_* (*t*) is the time coordinate of the first bending eigenmode in a vertical plan, and *X_c_*(*x*) stands for its eigenfunction.

(5) $$X_{c}\;(\;x\;)=\;\sin\;\beta \;x\;+\;\sinh\;\beta \;x\;-\frac{\sin\;\beta \;L_{c\;}-\;\sinh\;\beta L_{c}\;}{\cos\;\beta \;L_{c}\;-\cosh\;\beta \;L_{c}\;}\left(\;\cos\;\beta x+\cosh\;\beta x\right)$$

With $\beta \;=\sqrt[{4}]{w_{c}^{2}\;\rho_{c}\;/(E\;I)}$ and $\cos\beta L_{c}\cosh\beta L_{c}-1=0$.

When using the modal analysis approach and considering the eigenfunction’s orthogonality property in the vertical bending of a vehicle body, Equation (1) becomes three two-order deferential equations with ordinary derivatives, representing the bounce, pitch, and bending movements in the CB [40,41,42,43,44,45,46,47].

(6) $$m_{c}{\ddot{z}}_{c}=\sum_{i=1}^{2}F_{ci}+\sum_{k=1}^{n}F_{ek}$$

(7) $$J_{c}{\ddot{\theta }}_{c}=\sum_{i=1}^{2}F_{ci}\left(l_{i}-\frac{L_{c}}{2}\right)+\sum_{k=1}^{n}F_{ek}\left(l_{ek}-\frac{L_{c}}{2}\right)$$

(8) $$m_{mc}{\ddot{T}}_{c}+c_{mc}{\dot{T}}_{c}+k_{mc}T_{2}=\sum_{i-1}^{2}F_{ci}X_{c}(l_{i})+\sum_{k=1}^{n}F_{ek}X_{c}(l_{ek})$$

where $m_{mc},k_{mc}{,c}_{mc}$ are CB modal mass, damping, and stiffness, which are shown in Equation (9).

(9) $$k_{mc}=EI{\int_{0}^{L}\left(\frac{d^{2}X_{c}}{dx^{2}}\right)}^{2}dx,c_{mc}=\mu I{\int_{0}^{L}\left(\frac{d^{2}X_{c}}{dx^{2}}\right)}^{2}dx,m_{mc}=\rho_{c}\int_{0}^{L}X_{c}^{2}dx$$

### 2.2. Modeling of Bogies

For each bogie, a single mode of vibration is considered, namely bounce $z_{bi}$ with *i* = 1; 2. The pitch movement of the bogie is neglected since it is not transmitted to the vehicle’s CB in this model. The equations for the bounce movements of the bogies are

(10) $$m_{b}{\ddot{z}}_{b1}=\sum_{j=1}^{2}F_{bj}-F_{c1}$$

(11) $$m_{b}{\ddot{z}}_{b2}=\sum_{j=3}^{4}F_{bj}-F_{c2}$$

where $F_{bj}$ stands for the forces coming from the primary suspension corresponding to axle *j* as follows:

(12) $$F_{b1,2}=-2c_{b}({\dot{z}}_{b1}-{\dot{\eta }}_{1,2})-2k_{b}(z_{b1}-\eta_{1,2})\;for\;\begin{array}{l} j=1,2 \end{array}$$

(13) $$F_{b3,4}=-2c_{b}({\dot{z}}_{b2}-{\dot{\eta }}_{3,4})-2k_{b}(z_{b2}-\eta_{3,4})\;for\;\begin{array}{l} j=3,4 \end{array}$$

where $c_{b}$ (0.0589 MN-s/m) and $k_{b}$ (0.42375 MN/m) are the damping and stiffness coefficients of the primary suspension system. The suspended equipment has bounce movements that are described in the following equation:

(14) $$m_{ek}{\dot{z}}_{ek}=-\sum_{k=1}^{n}F_{ek}$$

The system equations can be written as the following

(15) $$M\;\ddot{p}+C\;\dot{p}+\mathrm{Kp}=P\ddot{\eta }+R\eta$$

The inertia, damping, and stiffness matrices are M, C, and K, respectively, while the track displacement and velocity input matrices are P and R.

## 3. Finite Element Analysis of Car Body

### 3.1. Finite Element Model of Car Body

**Figure 2:**
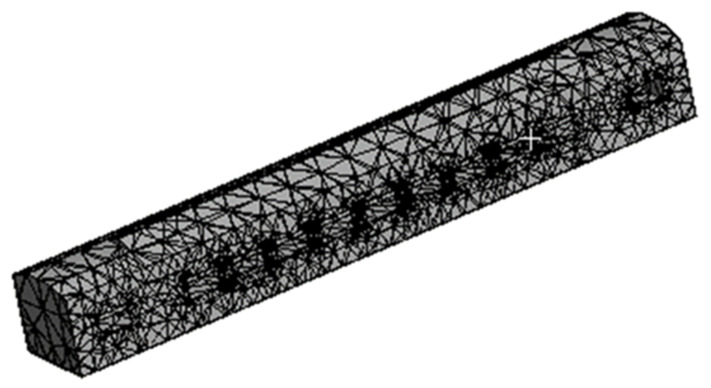
Mesh model of ICF passenger coach in ANSYS.

**Figure 3:**
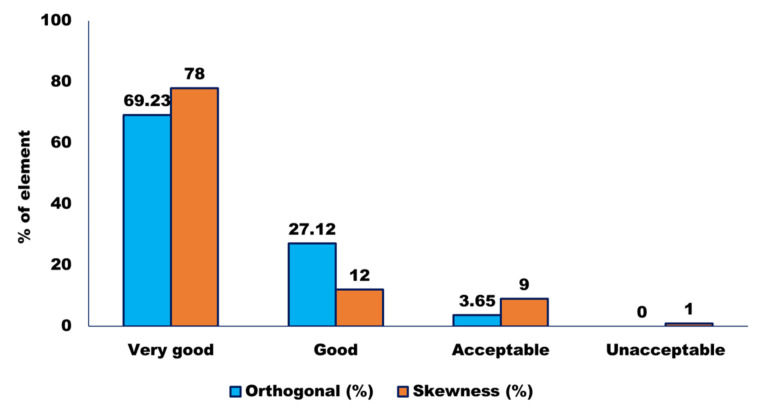
Mesh quality check using orthogonal and skewness quality.

A 3D finite element model of CB created in the ANSYS software (Figure 2) was used for modal analysis of the ICF vehicle. A total of 1658 shell elements and 1325 beam elements were used to discretize the geometry. The contact regions were identified: an interface between the car body and the center pivot top and an interface between the center pivot top and bottom. Moreover, other equipment are connected. There are a total of 598,273 nodes and 112,477 items in this structure. A mesh quality check employing orthogonal and skewness quality, as well as a mesh metric, is shown in Figure 3.

A mesh metric was validated using orthogonal quality and skewness quality. A significant (approximately 70%) number of the total elements lie in the ‘perfect’ zone, followed by the remaining (approximately 27%) number in the ‘good’ zone and a meager amount (approximately 3%) in the acceptable zone. A negligible number of elements fall in the ‘bad zone’, i.e., from 0.01–0.001. Since there are no elements in the ‘unacceptable zone’ of 0.001–0, the minimum value obtained is 0.0117. Thus, the mesh quality has passed the orthogonality test.

The skewness quality of the discretized elements is expressed in terms of the percentage of volume/area of the total elements. The skewness quality of nearly 50% of the elements is ‘excellent’. In contrast, nearly 28% of the elements have ‘very good’ skewness quality, followed by nearly 12% with a ‘good’ skewness quality tag. Nearly 9% are tagged as ones with ‘acceptable’ quality levels. A mere 1% fall into the category of ‘bad’, with highly negligible elements at an ‘unacceptable’ level of ‘1’. Thus, the skewness quality of the elements is to be hailed. The bulk of the elements is in the perfect zone, with only a minor percentage in the unpleasant zone, as can be shown. As a result, the mesh’s orthogonal and skewness quality tests were both passed.

### 3.2. Modal Analysis of the Car Body

Modal analysis is employed to solve the linear system’s motion equations without damping and to find the system’s natural frequency [48,49,50]. Analytical approaches could not precisely determine the natural frequency due to the complicated construction of the CB [37,51,52,53,54,55,56,57,58,59,60,61,62]. However, finite element methods might be used to solve it [3]. The solution of the free vibration of the system (See Equation (15)), ignoring the structural damping on the modal frequency, is given by:

(16) $$x=\delta \sin\left(\omega t+\phi_{0}\right)$$

where $\phi_{0}$ and *ω* are the initial phase and circular eigenfrequency, respectively, and *δ* is a magnitude vector. Equations (15) and (16) are solved to seek non-zero solutions. For such solutions,

(17) $$\left|K-\omega^{2}M\right|=0$$

The eigenvalues and corresponding eigenvectors of Equation (17) gave the fundamental modes of the system.

## 4. DVA Theory for Euler-Bernoulli Beam

The CB and suspended equipment formed a linked system, which varies from a single CB. It was critical to incorporate the additional mass, which may be considered DVA, while designing the CB as considered by Shi et al. [13]. Because the spring-mass model could not manage the flexible vibration of the CB, the Euler-Bernoulli beam model was adopted [20].

### 4.1. Modal Analysis of a Uniform Euler-Bernoulli Beam

**Figure 4:**
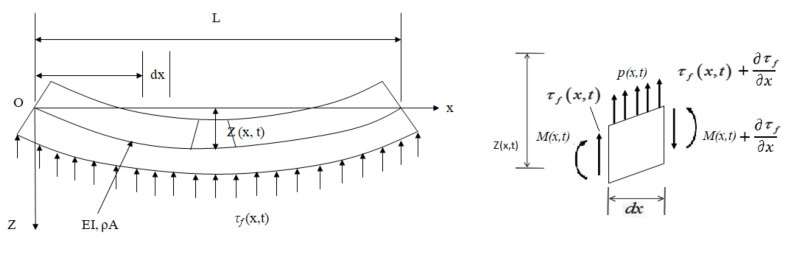
Euler-Bernoulli beam modal analysis [13].

In Figure 4, *L* is the length of the beam, *EI* represents the flexural rigidity, *ρA* is the mass per unit length in which *ρ* represents the density, and *A* represents the cross-sectional area [63]. The micro-element movement equation and the beam moment equilibrium equation are as follows.

(18) $$\left[\tau_{f}(x,t)+\frac{\partial \tau_{f}}{\partial x}dx\right]-\tau_{f}(x,t)+p(x,t)=\rho Adx\frac{\partial^{2}z}{\partial t^{2}}$$

(19) $$\left[M(x,t)+\frac{\partial M}{\partial x}dx\right]-M(x,t)+\left[\tau_{f}(x,t)+\frac{\partial \tau_{f}}{\partial x}dx\right]dx+p(x,t)dx\frac{dx}{2}=0$$

Equation (20) is considered to explain the proper differential equation for the uniform Euler beam:

(20) $$\frac{\partial^{2}}{\partial x^{2}}\left[EI\frac{\partial^{2}z(x,t)}{\partial x^{2}}\right]+\rho A\frac{\partial^{2}z(x,t)}{\partial t^{2}}=p(x,t)$$

The external force is zero for determining the eigenfrequencies, hence *p* (*x*, *t*) = 0. Assuming that the solution is harmonic,

(21) z(x,t)=Y(x)sin(ωt−α)

where *ω* was the angular frequency and *α* was some phase constant, for Equation (22) gives:

(22) $$\frac{\partial^{2}}{\partial x^{2}}\left[EI\frac{\partial^{2}Y(x)}{\partial x^{2}}\right]\sin(\omega t-\alpha )+\rho A(-\omega^{2})Y(x)\sin(\omega t-\alpha )=0$$

(23) $$\mathrm{Substituting}\;\beta^{4}=\frac{\rho A\omega^{2}}{EI}$$

The following fourth-order differential equation for the vibration of the beam was obtained by decreasing the sin (*ωt*-*α*) terms:

(24) $$\frac{d^{4}Y(x)}{dx^{4}}-\beta^{4}Y(x)=0$$

When *r* = ±*β* or *r* = ±*iβ*, a solution for Equation (24) in the form *Y*(*x*) = *e^rx^* would satisfy the differential equation [13]. Equation (24)’s general solution yielded the following shape function:

(25) Y(x)=Asinh(βx)+Bcosh(βx)+Csin(βx)+Dcos(βx)

The boundary conditions determine the eigenfrequencies. If *x*_0_ = 0 or *x*_0_ = *L*, the endpoints of the beam have no momentum nor shear force in the free-free situation.

(26) $$-EI{\left.\frac{\partial^{2}z(x,t)}{\partial x^{2}}\right|}_{x=x_{0}}=0,-\frac{\partial }{\partial x}{\left.\left[EI\frac{\partial^{2}z(x,t)}{\partial x^{2}}\right]\right|}_{x=x_{0}}=0$$

Boundary conditions could be written as:

(27) $$\left.\begin{array}{l} z^{\prime \prime }(0,t)=0\\ z^{\prime \prime }(L,t)=0\\ z^{\prime \prime \prime }(0,t)=0\\ z^{\prime \prime \prime }(L,t)=0 \end{array}\right\}\Rightarrow \left.\begin{array}{l} Y^{\prime \prime }(0)=0\\ Y^{\prime \prime }(L)=0\\ Y^{\prime \prime \prime }(0)=0\\ Y^{\prime \prime \prime }(L)=0 \end{array}\right\}$$

where the prime denotes a partial derivative of *x* and *L* denotes the length of the beam. The above equations become the following with Equation (25) inserted as:

(28) $$\;\left[\begin{array}{cccc} 0 & 1 & 0 & -1\\ \sinh(\beta L) & \cosh(\beta L) & -\sin(\beta L) & -\cos(\beta L)\\ 1 & 0 & -1 & 0\\ \cosh(\beta L) & \sinh(\beta L) & -\cos(\beta L) & \sin(\beta L) \end{array}\right]\left[\begin{array}{c} A\\ B\\ C\\ D \end{array}\right]=\left[\begin{array}{c} 0\\ 0\\ 0\\ 0 \end{array}\right]$$

By increasing the determinant provided in Equation (28), an algebraic equation known as the frequency equation was discovered. The frequency equation of the final form becomes the following after various simplifications.

(29) 1−cos(λ)cosh(λ)=0

where *λ* = *βL*. The lowest solution corresponds to a rigid CB movement since the beam was free-free; that is, floating in space. *λ*_1_ was the bending mode. The results might be broadly represented as:

(30) $$\lambda_{i}\approx \left(n+\frac{1}{2}\right)\pi ,n\geq 1$$

Combining Equations (25) and (28) yields the form function, which is described as:

(31) $$Y_{i}(x)=\cosh\beta_{i}x+\cos\beta_{i}x-\frac{\cosh\lambda_{i}x-\cos\lambda_{i}x}{\sinh\lambda_{i}x-\sin\lambda_{i}x}\left(\sinh\beta_{i}x+\sin\beta_{i}x\right)$$

where $\beta_{i}=\lambda_{i}/L$.

The beam’s normal modes are orthogonal and have been normalized in such a way that:

(32) $$\int_{0}^{L}Y_{i}(x)Y_{j}(x)dx=\left\{\begin{array}{l} L\\ 0 \end{array}\right.\left.\begin{array}{l} i=j\\ i\neq j \end{array}\right\}$$

It can be shown that:

(33) $$\int_{0}^{L}\left(\frac{d^{2}Y_{i}}{dx^{2}}\right)dx=\beta_{i}{}^{4}L$$

### 4.2. Optimum Suspension Frequency of DVA

**Figure 5:**
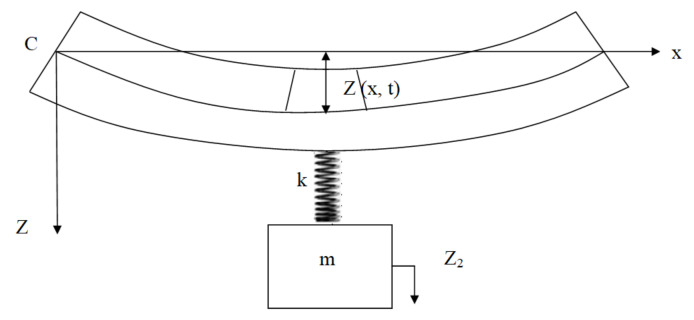
Model of the DVA applied on a flexible beam [13].

Based on the DVA principle, the equipment *m* was represented as a vibration absorber. The absolute coordinate of the motion of the absorber mass was *z*_2_, and the suspension stiffness was *k* [11,12,13,14]. The beam and a simple mass were then connected to form a coupled system, as shown in Figure 5. The distance between the suspended equipment and the car body end is assumed to be *x*, and the displacement is represented by *z*(*x*, *t*). Figure 5 depicts the overall model’s dimension and coordinate definition [13,39,64]. T and U denote the kinetic and potential energy of the linked system, respectively.


(34) $$\left.\begin{array}{l} T=\frac{1}{2}m{\dot{z}}_{2}+\frac{1}{2}\rho A\int_{0}^{L}{\dot{z}}^{2}(x,t)dx,\\ U=\frac{1}{2}k{\left[z(x,t)-z_{2}\right]}^{2}+\frac{1}{2}EI{\int_{0}^{L}\left(\frac{d^{2}z}{dx^{2}}\right)}^{2}dx \end{array}\right\}$$

The virtual work done by a harmonic externally force *p*(*x*) *e^iωt^* is:

(35) $$\delta W=\int_{0}^{L}p(x)e^{i\omega t}\delta zdx$$

Here, only one simple mode of beam flexure will be considered such that:

(36) $$z(x,t)=Y_{1}(x)z_{1}(t),$$

where *Y*_1_(*x*) is the classical beam mode associated with the first natural frequency of the beam, and $z_{1}\left(t\right)$ is the corresponding modal coordinate. Substitution of Equation (36) into Equations (34) and (35) gives the appropriate differential equation of *T*, *U*, and *δW* as

(37) $$\left.\begin{array}{l} T=\frac{1}{2}m{{\dot{z}}_{2}}^{2}+\frac{1}{2}\rho {Az_{1}}^{2}\int_{0}^{L}{Y_{1}}^{2}(x)dx,\\ U=\frac{1}{2}k{\left[Y_{1}\left(x\right)z_{1}-z_{2}\right]}^{2}+\frac{1}{2}{EIz_{1}}^{2}\int_{0}^{L}\left(\frac{d^{2}Y_{1}}{dx^{2}}\right)dx,\\ \delta W=\delta z_{1}\int_{0}^{L}p(x)Y_{1}(x)dxe^{i\omega t} \end{array}\right\}$$

where *ε* indicates the strength constant of the vertical force:

(38) $$\varepsilon =\frac{1}{2}m{\dot{z}}_{2}{}^{2}+\frac{1}{2}\rho ALz_{1}{}^{2}$$

Moreover, using Equations (32), (33) and (38), the integrals of Equation (37) can be evaluated so that the energies become:

(39) $$\left.\begin{array}{l} T=\frac{1}{2}m{\dot{z}}^{2}{}_{2}+\frac{1}{2}\rho ALz_{1}{}^{2},\\ U=\frac{1}{2}k{\left[Y_{1}(x)z_{1}-z_{z}\right]}^{2}+\frac{1}{2}EI\beta_{1}{}^{4}Lz_{1}{}^{2},\\ \delta W=\varepsilon e^{i\omega t}\delta z_{1} \end{array}\right\}$$

If *z*_1_ and *z*_2_ are considered generalized coordinates, and their associated generalized forces are designated as $\;\tau_{f1}$ and $\tau_{f2}$, then:

(40) $$\delta W=\tau_{f}{}_{1}\delta z_{1}+\tau_{f}{}_{2}\delta z_{2}$$

When Equation (40) is compared to Equation (39), the equation of generalized force is denoted as:

(41) $$\tau_{f}{}_{1}=\varepsilon e^{i\omega t},\tau_{f}{}_{2}=0$$

The coupled system’s Lagrange equation is written as:

(42) $$\Gamma =T-U=\frac{1}{2}m{\dot{z}}_{2}{}^{2}+\frac{1}{2}\rho AL{\dot{z}}_{1}{}^{2}-\frac{1}{2}k{\left[Y_{1}(x)z_{1}-z_{2}\right]}^{2}-\frac{1}{2}EI\beta_{1}{}^{4}Lz_{1}{}^{2}$$

Substitution of relations Γ, $\tau_{f1}$, and $\tau_{f2}$ into the second Lagrange’s equation gives:

(43) $$\frac{d}{dx}\left(\frac{\partial \Gamma }{\partial {\dot{z}}_{i}}\right)-\frac{\partial \Gamma }{\partial z_{i}}=\tau_{f}{}_{i},i=1,2$$

Now, a set of differential equations is obtained in matrix form:

(44) $$\left(\begin{array}{cc} \rho AL & 0\\ 0 & m \end{array}\right)\left\{\begin{array}{c} {\ddot{z}}_{1}\\ {\ddot{z}}_{2} \end{array}\right\}+\left(\begin{array}{cc} kY_{1}{}^{2}(x)+EI\beta_{1}{}^{4}L & -kY_{1}(x)\\ -kY_{1}(x) & k \end{array}\right)x\left\{\begin{array}{c} z_{1}\\ z_{2} \end{array}\right\}=\left\{\begin{array}{c} \varepsilon \\ 0 \end{array}\right\}e^{i\omega t}$$

The amplitude-frequency characteristics *R* (*ω*) might be determined between the generalized coordinate’s $z_{1}$ and the generalized force $\tau_{f1}=\varepsilon e^{i\omega t}$ as a result of the vibration analysis of amplitude-frequency characteristics of discrete vibration systems.

(45) $$R(\omega )=\frac{k-m\omega^{2}}{\rho ALm\omega^{4}-\omega^{2}[\rho ALk+mkY_{1}{}^{2}(x)+mEI\beta_{1}{}^{4}L]+kEI\beta_{1}{}^{4}L}$$

To generalize the analysis, dimensionless quantities can be defined as follows.

$$\mathrm{Dimensionless}\;\mathrm{frequency}\;g^{2}=\omega^{2}/\left(\frac{EI\beta_{1}^{4}}{\rho A}\right)\;\mathrm{Mass}\;\mathrm{ratio}\;\;\;u=m/\rho AL\;\mathrm{Tuning}\;\mathrm{ratio}\;\;\;\;\;f^{2}=\left(\frac{k}{m}\right)/\left(\frac{EI\beta_{1}^{4}}{\rho A}\right)$$

Equation (45) may be replaced with these dimensionless parameters to get the dynamic amplification factor of generalized coordinate *z*_1_ to generalized force *τ*_1_.

(46) $$A(g)=\frac{1}{EI\beta_{1}{}^{4}L}{\left[\frac{f^{2}-g^{2}}{g^{4}-g^{2}\{1+f^{2}[1+uY_{1}{}^{2}(x)]\}+f^{2}}\right]}_{\;}$$

The exception that the mass ratio has been substituted by $uy_{1}^{2}$. (*x*), the approximate frequency response function described in Equation (46), was of the same shape as that studied by Snowdon [65]. The optimal tuning ratio in this scenario is as follows:

(47) $$f_{opt}=\frac{1}{1+uY_{1}^{2}\left(x\right)}$$

## 5. Validation of Numerical Modeling

**Figure 6:**
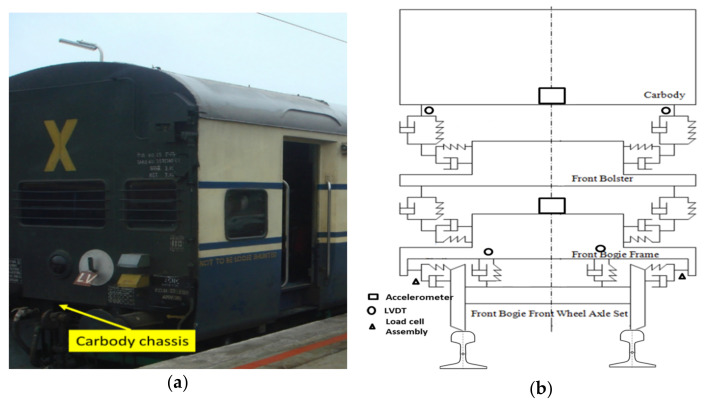
Field test line diagram of ICF coach. (**a**) ICF coach; (**b**) sensor location.

An on-track vibration test was designed and carried out to understand the vibration transition between the two suspension systems and the coupled vibrating between the car body and its suspended equipment. Accelerations on the wheelset axle-box, bogie frame, and connections between the car body and its suspended equipment were measured through accelerometers and displacement sensors connected to the data acquisitions system in the equipment cabin through cables [66,67,68,69]. The on-track vibration test was designed for long-term and continuous vehicle dynamics recording. Based on the long-term dynamic’s performance test for the prototype coach, the vibration characteristics of the equipment are analyzed and shown in Figure 6. The lateral and vertical accelerations of the equipment and the car body under different bogie running performances are measured, and then the spectrum composition and the vibration source are analyzed. The rolling stock is run on a nominated section of the IR track between Mathura and Palwal at maximum speeds of 10% higher than operating speeds. The oscillation trials were performed on a maintained track at speeds ranging from 110 to 180 km/h, respectively [70,71,72,73,74].

The sampling frequency of the frame acceleration is 2 kHz, and the passband frequency range is 0.5–12 Hz. The sampling frequency of the equipment acceleration is 1 kHz, and the passband frequency range is 0.1–200 Hz. The numerical model is validated through the oscillation trial conducted by RDSO on the actual condition. In particular, the ride comfort for passengers of the proposed numerical passive model is compared with the experimental results obtained by RDSO. The oscillation trials are on track tests with a prototype coach equipped and instrumented for recording acceleration, displacement, speed, and events.

**Figure 7:**
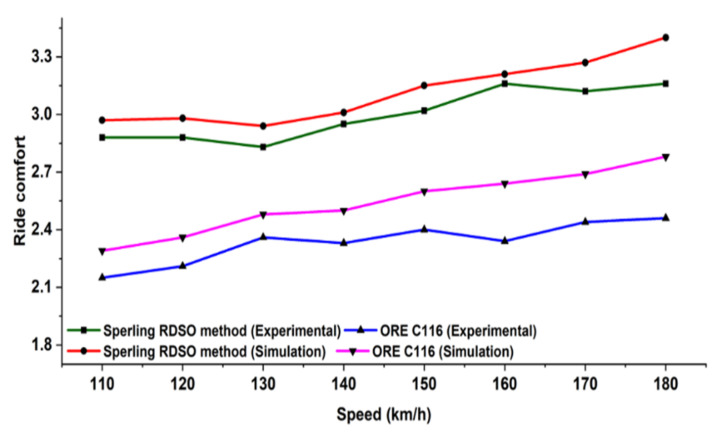
Comparison of numerical and experimental analysis for loaded conditions.

During oscillation trials, the maximum vertical and lateral ride index in empty and loaded conditions were found. The ride index was calculated with ORE C 116 and RDSO methods. The value of the ride index as per ORE C 116 was less than 2.75 and 3.25 (preferable) or 3.50 maximum as per RDSO criteria. The riding behavior of the same was found satisfactory. However, the ride index values need to be improved. Numerical simulation is performed using both the methods, i.e., ORE C 116 and RDSO Sperling index, by considering the RDSO test track condition for empty and loaded vehicles. Figure 7 shows the comparison between the experimental and numerical ride index under empty and fully loaded conditions, respectively. Moreover, it can be seen that the results obtained from the numerical analysis of the proposed model have reached a good overall agreement with the experimentally measured results. The variation in the ride index is mainly due to the elements not considered to reduce the complexity of the mathematical model.

## 6. Result and Discussion

A passenger rail vehicle CB consists of different equipment suspended under the chassis, consisting of tons of kilograms. Hence the relationship between suspended equipment and CB modes is developed. The vertical motion equations of the coupled system were given in Section 2 and are considered to analyze the response of the system and the influence of suspended equipment parameters on the CB modes.

### 6.1. Car Body Modes

**Figure 8:**
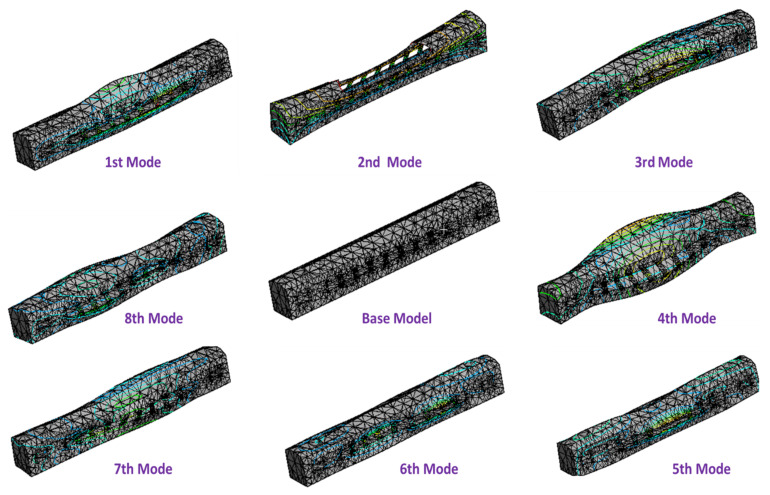
Modal frequency and mode shapes for passenger car body of ICF coach.

The influence of suspension parameters on the modal vibration of the CB was analyzed using FEA and MATLAB. Both the rigid and elastic suspension cases were investigated to depict the vibration regularity clearly and intelligibly. The rigid way of attaching types of equipment to the CB could be performed by altering or welding the hardware underneath the CB. Moreover, the flexible way is to utilize detachment segments, such as the rubble component, to join these two sections. The deformation of suspended equipment was then obtained using FEA for rigid and flexible structures. The eigenmode obtained from the finite element model of the passenger CB showed the deformation at each mode given in Figure 8. The deformation of the CB was mainly affected by its frame and side walls and its suspension parameters. The comfort level and stability of a passenger coach were affected by its deflections. Mode shape was obtained up to a frequency of 25 Hz. The various mode shapes and corresponding frequencies are shown in Table 1.

### 6.2. Optimum Suspension Frequency of Equipment’s

**Figure 9:**
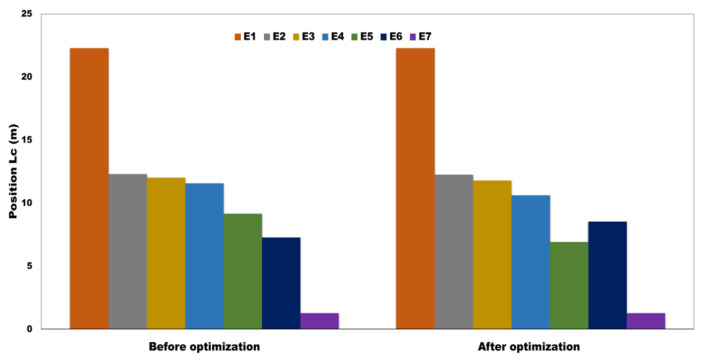
Location of the equipment’s comparison from the CB center (*Lc*) before optimization and after optimization.

The CB chassis has various kinds of equipment suspended, i.e., waste discharge unit- I (E1), battery Box (E2), transformer (E3), braking Unit (E4), water Tank (E5), pump equipment (E6), and waste discharge unit (II) (E7). Their masses and positions were different and connected to the CB chassis via rubber elements whose properties were discrepant for various equipment. The stiffness of rubber elements would affect the suspension frequency directly, which should be designed according to Equation (47), and the shape function of the CB could be calculated from Equation (25). The location of the equipment’s comparison from the CB center (*Lc*) before and after optimization is shown in Figure 9.

**Figure 10:**
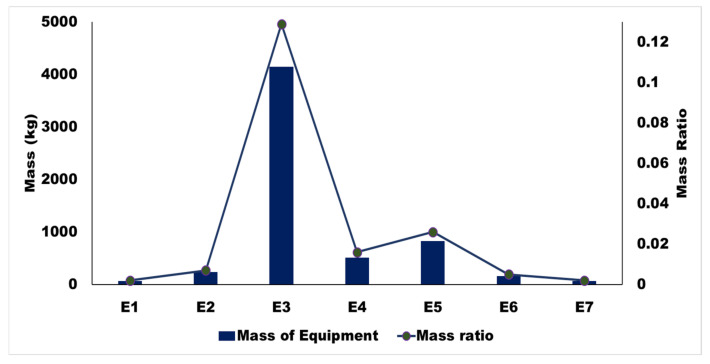
Mass and mass ratio of the suspended equipment.

Before the calculation, the FEM was applied to obtain the modal modes of the CB without any equipment mounted. For example, taking an ICF passenger coach, there were mainly five mounted devices on the chassis with assorted positions. The devices weigh about 4.15 to 0.16 tons (see Figure 10), varying from the heaviest to the lightest and mass ratio, shape function, and optimized position from CB center and frequency ratio and optimum frequency of different equipment from E1 to E7. It was found that equipment suspension frequencies were close to the bending mode frequencies of a CB.

### 6.3. Effect of Suspension Equipment on Car Body Transmissibility and Vertical Bending Frequency

**Figure 11:**
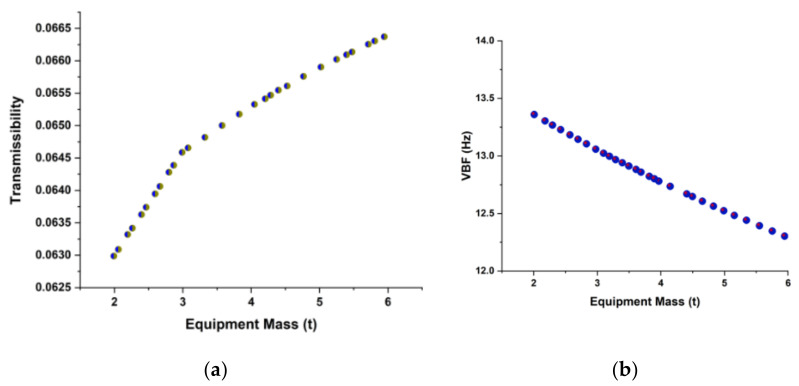
Elastic suspended equipment effect on CB mode due to equipment mass. (**a**) Transmissibility, (**b**) VBF.

The effect of suspended equipment, i.e., elastic, on CB mode is evaluated and shown in Figure 11a,b. The rigid suspended equipment is equal to increasing the mass of the CB without reducing its rigidity. This reduces the first-order bending frequency while increasing transmissibility. As the suspension frequency rises, so does the bending frequency, and the low-order bending frequency approaches that of the absence of suspended equipment. The high-order bending frequency approaches infinity and finally equals the bending frequency under stiff suspension. However, when the suspension frequency changes, so does the transmissibility. When the suspension frequency increases, the LF transmissibility increases while the high-frequency transmissibility decreases.

### 6.4. Effect on Car Body Mode Due to the Optimal Frequency of Suspended Equipment

**Figure 12:**
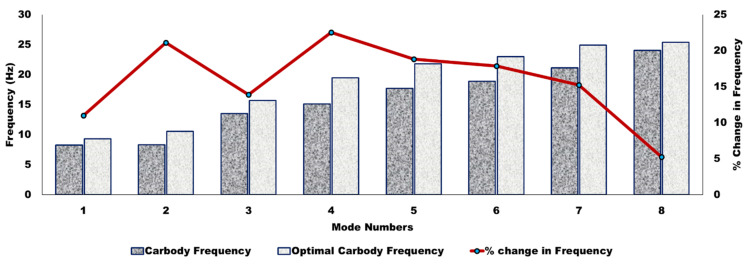
Effect of suspension parameter on the modal frequency of a CB.

The dominating elastic deformation occurred up to a frequency range of 20 Hz. Hence, it was crucial to optimize these modes to get the better ride quality of the vehicle and ride comfort for human passengers. The relationship was developed between CB modal frequency and suspension parameters of underframe equipment to optimize those. The optimum suspension frequency of the equipment for suspension parameters is shown in Figure 12. The effect of that suspension parameter played a significant role in reducing the flexible vibration of the CB. Heavy equipment was mounted in the center and followed by light equipment, as heavy equipment played an essential role in surpassing the vibration. However, since excitation frequencies were likely to be in the range of 5–9 Hz, the modal analysis revealed two frequencies, i.e., 8.64 and 8.79 Hz. Moreover, the third mode was a vertical mode at a frequency of 12.14 Hz. Those modes showed a possibility of resonance in the vertical direction. Hence, the frequency needs to alter away from 5–9 Hz to avoid resonance. DVA equipment was optimized to improve the model frequency of the 1st, 2nd, and 3rd modes from 8.64 to 9.57 Hz, 8.79 to 10.63 Hz, and 12.14 to 14.06 Hz, respectively, to avoid the possibility of resonance, as shown in Figure 12. Moreover, it concluded that the % change in the model frequency of CB due to suspended equipment was about 13.65% for vertical bending (mode 3).

Optimizing the DVA parameters showed an effect on avoidance of the vibrating resonance. Moreover, the optimization showed a change of about 9.71% in the first mode and 17.30% in the second mode of diagonal distortion and shell breathing in a lateral direction, respectively. A significant effect of 13.65% in mode frequency improvement is seen in the vertical mode. Similarly, improvement in the flexible variation was obtained by optimizing various equipment locations under the CB frame; about 23.68%, 26.87%, and 25.68% of the fourth, fifth, and sixth modes, respectively. Those optimized locations give aid in designing lighter structures for the passenger CB.

## 7. Conclusions

This paper presented a rigid-flexible 3D rail vehicle model that analyzed the impacts of different equipment suspended under the chassis based on the CB mode’s mass, location, and frequency. The proposed model was applied to study the effects of car body flexibility on the dynamic performances of a rail vehicle. Moreover, the rigid-flexible rail vehicle model was validated by experimental investigation. The analysis shows that equipment with a considerable mass should be suspended near the center of the CB to optimize the frequency of high-frequency bending. The optimization of DVA shows a significant improvement in terms of resonance, i.e., 9.71% for the first mode, 17.30% for the second mode, and 13.65% for the first vertical mode. The weight of the equipment has a significant impact on the first bending frequency. The frequency of heavy, hanging equipment should be low enough to promote high-frequency transmissibility and improve vibration characteristics. The frequency of suspended equipment should be lower than the bending frequency of the CB.

## Figures and Tables

**Table 1:** Modal frequency and mode shapes for passenger CB with the initial location of the equipment.

Mode Number	Mode Shape Description	Frequency (Hz)	Effect
1	Lateral swaying of sidewalls	8.64	It affects the lateral direction due to the deflection of sidewalls
2	Rhombic mode or diagonal distortion	8.79	It affects the sidewall via displacement due to excitation frequencies
3	Vertical bending mode	12.14	It is an effect in the vertical vibration of the CB
4	Lateral shell breathing with rear and front walls swaying	14.25	It affects the end and shell of the longitudinal walls was also breathing, which caused an opposed motion
5	Lateral and rolling swaying at central length	16.87	It affects the rolling motion of the CB
6	Torsion and longitudinal diagonal distortion of front wall	19.87	Coupled lateral and longitudinal vibration together
7	Torsion and longitudinal diagonal distortion of rear wall	20.24	Coupled lateral and longitudinal vibration together
8	Shell breathing in longitudinal and lateral directions	22.14	A high rolling motion was experienced by a CB

## Data Availability

Not applicable.

## References

[1] Smith C.C., Gilchrist A.J., Wormley D.N. (1975). Multiple and Continuous Span Elevated Guideway-Vehicle Dynamic Performance. J. Dyn. Syst. Meas. Control.

[2] Chen C., Wang K. (2006). Study on modeling of lateral semi-active suspension system of high-speed train. J. Vib. Shock.

[3] Sharma S.K., Mohapatra S., Sharma R.C., Alturjman S., Altrjman C., Mostarda L., Stephan T. (2022). Retrofitting Existing Buildings to Improve Energy Performance. Sustainability.

[4] Wu Q., Cole C., Spiryagin M., Chang C., Wei W., Ursulyak L., Shvets A., Murtaza M.A., Mirza I.M., Zhelieznov K. (2021). Freight train air brake models. Int. J. Rail Transp..

[5] Vishwakarma P.N., Mishra P., Sharma S.K. (2021). Characterization of a magnetorheological fluid damper a review. Mater. Today Proc..

[6] Lee J., Han J., Sharma S.K., Joshi P., Gupta S.S., Shukla A.K., Gautam S.S. (2021). Structural Analysis on the Separated and Integrated Differential Gear Case for the Weight Reduction. Advances in Engineering Design, Proceedings of the 2nd International Conference on Future Learning Aspects of Mechanical Engineering (FLAME), Noida, India, 5–7 August 2020.

[7] Sharma R.C., Palli S., Sharma N., Sharma S.K. (2021). Ride Behaviour of a Four-wheel Vehicle using H Infinity Semi-Active Suspension Control under Deterministic and Random Inputs. Int. J. Veh. Struct. Syst..

[8] Sharma S.K., Sharma R.C., Lee J. (2021). In situ and experimental analysis of longitudinal load on carbody fatigue life using nonlinear damage accumulation. Int. J. Damage Mech..

[9] Sharma S.K., Sharma R.C., Lee J. (2021). Effect of Rail Vehicle-Track Coupled Dynamics on Fatigue Failure of Coil Spring in a Suspension System. Appl. Sci..

[10] Sharma R.C., Sharma S., Sharma N., Sharma S.K. (2022). Linear and Nonlinear Analysis of Ride and Stability of a Three-Wheeled Vehicle Subjected to Random and Bump Inputs Using Bond Graph and Simulink Methodology. SAE Int. J. Commer. Veh..

[11] Gong D., Zhou J., Sun W., Sun Y., Xia Z. (2017). Method of multi-mode vibration control for the carbody of high-speed electric multiple unit trains. J. Sound Vib..

[12] Gong D., Zhou J., Sun W. (2013). On the resonant vibration of a flexible railway car body and its suppression with a dynamic vibration absorber. J. Vib. Control.

[13] Shi H., Luo R., Wu P., Zeng J., Guo J. (2014). Application of DVA theory in vibration reduction of carbody with suspended equipment for high-speed EMU. Sci. China Technol. Sci..

[14] Guo J., Shi H., Luo R., Wu P. (2019). Parametric Analysis of the Car Body Suspended Equipment for Railway Vehicles Vibration Reduction. IEEE Access.

[15] Tomioka T., Takigami T., Suzuki Y. (2006). Numerical analysis of three-dimensional flexural vibration of railway vehicle car body. Veh. Syst. Dyn..

[16] Sun W., Zhou J., Gong D., You T. (2016). Analysis of modal frequency optimization of railway vehicle car body. Adv. Mech. Eng..

[17] Diana G., Cheli F., Collina A., Corradi R., Melzi S. (2002). The Development of a Numerical Model for Railway Vehicles Comfort Assessment through Comparison with Experimental Measurements. Veh. Syst. Dyn..

[18] Sharma S.K., Lee J. (2021). Crashworthiness Analysis for Structural Stability and Dynamics. Int. J. Struct. Stab. Dyn..

[19] Sharma R.C., Sharma S., Sharma S.K., Sharma N., Singh G. (2021). Analysis of bio-dynamic model of seated human subject and optimization of the passenger ride comfort for three-wheel vehicle using random search technique. Proc. Inst. Mech. Eng. Part K J. Multi-Body Dyn..

[20] Sharma S.K., Phan H., Lee J. (2020). An Application Study on Road Surface Monitoring Using DTW Based Image Processing and Ultrasonic Sensors. Appl. Sci..

[21] Sharma S.K., Sharma R.C., Sharma N. (2020). Combined Multi-Body-System and Finite Element Analysis of a Rail Locomotive Crashworthiness. Int. J. Veh. Struct. Syst..

[22] Lee J., Sharma S.K. (2020). Numerical Investigation of Critical Speed Analysis of High-speed Rail Vehicle. Korean Soc. Precis. Eng..

[23] Bhardawaj S., Sharma R.C., Sharma S.K. (2020). Development in the modeling of rail vehicle system for the analysis of lateral stability. Mater. Today Proc..

[24] Sharma R.C., Sharma S., Sharma S.K., Sharma N. (2020). Analysis of generalized force and its influence on ride and stability of railway vehicle. Noise Vib. Worldw..

[25] Sharma S.K., Lee J. (2020). Finite Element Analysis of a Fishplate Rail Joint in Extreme Environment Condition. Int. J. Veh. Struct. Syst..

[26] Sharma R.C., Sharma S.K., Palli S. (2020). Linear and Non-Linear Stability Analysis of a Constrained Railway Wheelaxle. Int. J. Veh. Struct. Syst..

[27] Sharma S., Sharma R.C., Sharma S.K., Sharma N., Palli S., Bhardawaj S. (2020). Vibration Isolation of the Quarter Car Model of Road Vehicle System using Dynamic Vibration Absorber. Int. J. Veh. Struct. Syst..

[28] Bhardawaj S., Sharma R.C., Sharma S.K. (2020). Development of multibody dynamical using MR damper based semi-active bio-inspired chaotic fruit fly and fuzzy logic hybrid suspension control for rail vehicle system. Proc. Inst. Mech. Eng. Part K J. Multi-Body Dyn..

[29] Palli S., Sharma R.C., Sharma S.K., Chintada V.B. (2020). On methods used for setting the curve for railway tracks. J. Crit. Rev..

[30] Palli S., Sharma R.C., Sharma S.K., Muddada V., Sharma N. (2020). A case study on noise pollution and its effects. J. Crit. Rev..

[31] Sharma S.K., Lee J. (2020). Design and Development of Smart Semi Active Suspension for Nonlinear Rail Vehicle Vibration Reduction. Int. J. Struct. Stab. Dyn..

[32] Lavania S., Nagaria D. Eigen spectrum based moment matching technique for model order reduction. Proceedings of the 2015 39th National Systems Conference (NSC).

[33] Sharma R.C., Sharma S.K., Sharma N., Sharma S. (2020). Analysis of ride and stability of an ICF railway coach. Int. J. Veh. Noise Vib..

[34] Bhardawaj S., Sharma R., Sharma S. (2020). Ride Analysis of Track-Vehicle-Human Body Interaction Subjected to Random Excitation. J. Chin. Soc. Mech. Eng..

[35] Acharya A., Gahlaut U., Sharma K., Sharma S.K., Vishwakarma P.N., Phanden R.K. (2020). Crashworthiness Analysis of a Thin-Walled Structure in the Frontal Part of Automotive Chassis. Int. J. Veh. Struct. Syst..

[36] Bhardawaj S., Sharma R.C., Sharma S.K. (2020). Analysis of frontal car crash characteristics using ANSYS. Mater. Today Proc..

[37] Sharma S.K., Kumar A. (2017). Ride performance of a high speed rail vehicle using controlled semi active suspension system. Smart Mater. Struct..

[38] Dumitriu M. (2020). Numerical study of the influence of suspended equipment on ride comfort in high-speed railway vehicles. Sci. Iran..

[39] Lavania S., Nagaria D. Fminsearch Optimization Based Model Order Reduction. Proceedings of the IEEE 2016 Second International Conference on Computational Intelligence & Communication Technology (CICT).

[40] Sharma S.K. (2019). Multibody analysis of longitudinal train dynamics on the passenger ride performance due to brake application. Proc. Inst. Mech. Eng. Part K J. Multi-Body Dyn..

[41] Choppara R.K., Sharma R.C., Sharma S.K., Gupta T. (2019). Aero dynamic cross wind analysis of locomotive. IOP Conf. Ser. Mater. Sci. Eng..

[42] Bhardawaj S., Chandmal Sharma R., Kumar Sharma S. (2019). A Survey of Railway Track Modelling. Int. J. Veh. Struct. Syst..

[43] Lavania S., Nagaria D. (2019). Reduced Order Modeling of Linear Time-Invariant Systems Using Soft Computing Technique. Advances in Intelligent Systems and Computing.

[44] Goyal S., Anand C.S., Sharma S.K., Sharma R.C. (2019). Crashworthiness analysis of foam filled star shape polygon of thin-walled structure. Thin-Walled Struct..

[45] Lee J., Ngo V., Phan H., Nguyen T., Dao D.K., Cho Y. (2019). Scattering of Surface Waves by a Three-Dimensional Cavity of Arbitrary Shape: Analytical and Experimental Studies. Appl. Sci..

[46] Goswami B., Rathi A., Sayeed S., Das P., Sharma R.C., Sharma S.K. (2019). Optimization Design for Aerodynamic Elements of Indian Locomotive of Passenger Train. Advances in Engineering Design, Proceedings of the 2nd International Conference on Future Learning Aspects of Mechanical Engineering (FLAME), Noida, India, 5–7 August 2020.

[47] Bhardawaj S., Sharma R.C., Sharma S.K. (2019). Development and advancement in the wheel-rail rolling contact mechanics. IOP Conf. Ser. Mater. Sci. Eng..

[48] Sun Y., Gong D., Zhou J. (2016). Study on Vibration Reduction Design of Suspended Equipment of High Speed Railway Vehicles. J. Phys. Conf. Ser..

[49] Shi X., Zhu S., Ni Y.Q., Li J. (2018). Vibration suppression in high-speed trains with negative stiffness dampers. Smart Struct. Syst..

[50] Sun Y., Zhou J., Gong D., Sun W., Xia Z. (2017). A New Vibration Absorber Design for Under-Chassis Device of a High-Speed Train. Shock Vib..

[51] Palli S., Koona R., Sharma S.K., Sharma R.C. (2018). A Review on Dynamic Analysis of Rail Vehicle Coach. Int. J. Veh. Struct. Syst..

[52] Sharma S.K., Kumar A. (2017). Impact of electric locomotive traction of the passenger vehicle Ride quality in longitudinal train dynamics in the context of Indian railways. Mech. Ind..

[53] Dao D.K., Ngo V., Phan H., Pham C.V., Lee J., Bui T.Q. (2020). Rayleigh wave motions in an orthotropic half-space under time-harmonic loadings: A theoretical study. Appl. Math. Model..

[54] Park J., Lee J., Min J., Cho Y. (2020). Defects Inspection in Wires by Nonlinear Ultrasonic-Guided Wave Generated by Electromagnetic Sensors. Appl. Sci..

[55] Sharma R.C., Palli S., Sharma S.K., Roy M. (2017). Modernization of railway track with composite sleepers. Int. J. Veh. Struct. Syst..

[56] Sharma R.C., Sharma S.K. (2018). Sensitivity analysis of three-wheel vehicle’s suspension parameters influencing ride behavior. Noise Vib. Worldw..

[57] Sharma S.K., Kumar A. (2018). Disturbance rejection and force-tracking controller of nonlinear lateral vibrations in passenger rail vehicle using magnetorheological fluid damper. J. Intell. Mater. Syst. Struct..

[58] Sharma S.K., Kumar A. (2018). Ride comfort of a higher speed rail vehicle using a magnetorheological suspension system. Proc. Inst. Mech. Eng. Part K J. Multi-Body Dyn..

[59] Sharma S.K., Sharma R.C. (2018). Simulation of Quarter-Car Model with Magnetorheological Dampers for Ride Quality Improvement. Int. J. Veh. Struct. Syst..

[60] Sharma R.C., Sharma S.K., Palli S. (2018). Rail Vehicle Modelling and Simulation using Lagrangian Method. Int. J. Veh. Struct. Syst..

[61] Sharma S.K., Sharma R.C. (2018). An Investigation of a Locomotive Structural Crashworthiness Using Finite Element Simulation. SAE Int. J. Commer. Veh..

[62] Sharma S.K., Kumar A. (2018). Impact of Longitudinal Train Dynamics on Train Operations: A Simulation-Based Study. J. Vib. Eng. Technol..

[63] Lavania S., Nagaria D. (2016). BAT algorithm for model order reduction. Int. J. Math. Model. Numer. Optim..

[64] Sun Q., Wen Y., Zou Y. (2019). Study on the Vibration Suppression Method of Urban Railway Vehicles Based on a Composite Dynamic Vibration Absorber. MATEC Web Conf..

[65] Snowdon J.C. (1959). Steady-State Behavior of the Dynamic Absorber. J. Acoust. Soc. Am..

[66] Sharma S.K., Sharma R.C., Kumar A., Palli S. (2015). Challenges in Rail Vehicle-Track Modeling and Simulation. Int. J. Veh. Struct. Syst..

[67] Sharma S.K., Chaturvedi S. (2016). Jerk analysis in rail vehicle dynamics. Perspect. Sci..

[68] Sharma S.K., Kumar A. (2016). Dynamics Analysis of Wheel Rail Contact Using FEA. Procedia Eng..

[69] Fodor K.F., Chalivendra V., Kim Y.K., Lewis A.F. (2019). Dynamic mechanical behavior of flocked layer composite materials. Compos. Struct..

[70] Sankaran K.B. (2001). Maintenance Manual for Indian Railway.

[71] Krawler A. (2013). Air Brake System. https://www.sgi.sk.ca/air-brake/-/knowledge_base/air-brake/system-components.

[72] Tupe A.R. (2013). Handbook on Maintenance of Air Brake System in LHB Coaches (FTIL Type).

[73] Lavania S., Nagaria D. Pade approximation based moment matching technique for model order reduction. Proceedings of the 2015 International Conference on Computer, Communication and Control (IC4).

[74] Misra R.K. (2016). Technical Specification of Hot Coiled Helical Springs Used in Locomotives.

